# Quantitative comparison of automatic and manual IMRT optimization for prostate cancer: the benefits of DVH prediction

**DOI:** 10.1120/jacmp.v16i2.5204

**Published:** 2015-03-08

**Authors:** Yun Yang, Taoran Li, Lulin Yuan, Yaorong Ge, Fang‐Fang Yin, W. Robert Lee, Q. Jackie Wu

**Affiliations:** ^1^ Duke University Medical Center Durham NC USA; ^2^ The University of North Carolina at Charlotte Charlotte NC USA

**Keywords:** IMRT, optimization, automatic planning

## Abstract

A recent publication indicated that the patient anatomical feature (PAF) model was capable of predicting optimal objectives based on past experience. In this study, the benefits of IMRT optimization using PAF‐predicted objectives as guidance for prostate were evaluated. Three different optimization methods were compared. 1) Expert Plan: Ten prostate cases (16 plans) were planned by an expert planner using conventional trial‐and‐error approach started with institutional modified OAR and PTV constraints. Optimization was stopped at 150 iterations and that plan was saved as Expert Plan. 2) Clinical Plan: The planner would keep working on the Expert Plan till he was satisfied with the dosimetric quality and the final plan was referred to as Clinical Plan. 3) PAF Plan: A third sets of plans for the same ten patients were generated fully automatically using predicted DVHs as guidance. The optimization was based on PAF‐based predicted objectives, and was continued to 150 iterations without human interaction. $D_{\text{MAX}}$ and $D_{98\%}$ for PTV, $D_{\text{MAX}}$ for femoral heads, $D_{\text{MAX}}$, D_10cc_, $D_{25\%}/D_{17\%}$, and $D_{40\%}$ for bladder/rectum were compared. Clinical Plans are further optimized with more iterations and adjustments, but in general provided limited dosimetric benefits over Expert Plans. PTV $D_{98\%}$ agreed within 2.31% among Expert, Clinical, and PAF plans. Between Clinical and PAF Plans, differences for $D_{\text{MAX}}$ of PTV, bladder, and rectum were within 2.65%, 2.46%, and 2.20%, respectively. Bladder D_10cc_ was higher for PAF but <1.54% in general. Bladder $D_{25\%}$ and $D_{40\%}$ were lower for PAF, by up to 7.71% and 6.81%, respectively. Rectum D_10cc_, $D_{17\%}$, and $D_{40\%}$ were 2.11%, 2.72%, and 0.27% lower for PAF, respectively. $D_{\text{MAX}}$ for femoral heads were comparable (<35 Gy on average). Compared to Clinical Plan (Primary+Boost), the average optimization time for PAF plan was reduced by 5.2 min on average, with a maximum reduction of 7.1 min. Total numbers of MUs per plan for PAF Plans were lower than Clinical Plans, indicating better delivery efficiency. The PAF‐guided planning process is capable of generating clinical‐quality prostate IMRT plans with no human intervention. Compared to manual optimization, this automatic optimization increases planning and delivery efficiency, while maintaining plan quality.

PACS numbers: 87.55.D‐, 87.55.de, 87.53.Jw

## I. INTRODUCTION

Intensity‐modulated radiation therapy (IMRT) has been widely used to treat early stage prostate cancer and yields good clinic results. IMRT is well known for its ability of providing conformal coverage to the planning target volume (PTV), while sparing organs at risk (OARs) such as rectum, bladder, and femoral heads, in the case of prostate planning. In current clinical practice, IMRT planning for a specific patient is achieved by iteratively reducing dose to OARs in a trial‐and‐error fashion until the dose distribution is believed to be optimal for that patient. Lack of achievable patient‐specific OAR sparing information makes this manual approach time‐consuming.

From another perspective, with IMRT being implemented clinically for nearly two decades, experience and knowledge have been built and accumulated. Such expert knowledge has been implicitly built into each clinical approved plan created by expert radiation oncologists and planners. Learning from prior plans to predict patient‐specific optimal dose sparing is an innovative use of expert's knowledge.

Learning from prior plans has been reported previously by several research groups. Early attempts at predicting achievable dose sparing from prior plans have been more theoretical than practical.^(1)^ A number of recent methods have used prior plans to establish dose volume effects (i.e., predicting clinical outcomes based on delivered dose and other patient conditions.)^2^, ^3^, ^4^, ^5^ Other methods have employed prior plans for quality control,^6^, ^7^, ^8^, ^9^ and demonstrated that prior plan can serve as an important reference for improving and homogenizing the quality of new plans.^(10)^ More recently, predicting achievable dose‐volume histograms (DVHs) using prior knowledge has been reported.^9^, ^11^, ^12^ At our institution, Yuan et al.^(12)^ developed the patient anatomical features (PAF) based knowledge model, which estimates the OAR DVHs from prior plans by a stepwise multiple regression method. The PAF model was designed to predict the optimal achievable patient specific OAR DVHs. Such OAR DVHs can be further used to guide the IMRT planning/optimization process so that the trial‐and‐error process can be practically eliminated.

In this study we conducted a planning study with anonymized clinical cases. Each case is planned both manually by an experienced planner, and automatically under the guidance from the predicted DVHs. This study 1) evaluates the performance of the automatic prostate IMRT planning based on PAF model compared to human expert planning, and 2) quantifies the efforts required by human planner to achieve the optimal patient‐specific, organ‐sparing objectives.

## II. MATERIALS AND METHODS

Ten prostate cases were randomly selected and investigated in this study, including four low‐risk cases and six intermediate‐risk cases. IMRT plans were generated in Eclipse treatment planning system (v10.0, Varian Medical Systems, Palo Alto, CA). The clinical target volume (CTV) and OARs were contoured by attending physicians. The CTV included prostate alone for low‐risk prostate cancer and prostate plus seminal vesicles (SV) for intermediate‐risk prostate cancer. The planning target volume (PTV) was expanded from CTV with a 5 mm margin. Typical prescription dose at our institution was 2 Gy×38 fractions for low‐risk plans and 2 Gy×27 fractions (Primary,prostate+SV)+2 Gy×11 fractions (boost, prostate only) for intermediate‐risk plans. Sixteen plans were created for the four low‐risk cases and six intermediate‐risk cases in total. Seven coplanar 15 MV beams were used for primary plans with beam angle of 25°, 70°, 130°, 180°, 230°, 290°, and 335°, and five beams at 40°, 110°, 180°, 250°, and 320° for boost plans. All plans were normalized so that 100% prescription dose covers 95% of the PTV. Table 1 summarized the PTV, rectum, and bladder volume information for the ten cases studied, and also the overlaps between PTV and OARs, which is commonly considered as the limiting factor for OAR sparing in the high‐dose region.

**Figure 1:**
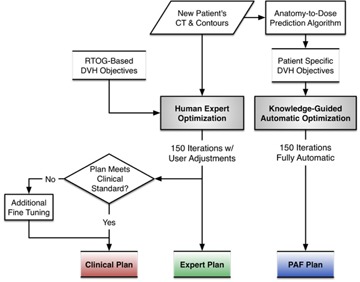
Study design showing the Expert Plan which involves 150 iterations with user adjustments. The planner decides whether to continue optimization after 150 iterations (Clinical Plan). PAF Plan uses DVHs predicted from PAF model and does not involve any user activities.

Each case in this study was planned using two different scenarios (human expert manual planning and DVH prediction‐guided automatic planning) and three types of plans were created (Expert Plan, Clinical Plan, and PAF Plan). The study design and planning flow are shown in Fig. 1. The patient CT images and structures such as body, PTVs, and OARs are used to predict the DVHs.^(12)^ The Expert Plan, Clinical Plan, and PAF Plan are then created with the beam angles mentioned above. The entire planning process was recorded by the screen capture software BB FlashBack (Blueberry Software, Birmingham, UK) for subsequent comparison and analysis.

**Table 1:** Patient anatomical information including volumes for PTV and ORAs and overlaps between PTV and OARs, which is commonly considered as the limiting factor for OAR sparing in the high‐dose region. Overlap is expressed in percentage of the OAR volume

		*PTV (cc)*	*Rectum (cc)*	*Overlap PTV & Rectum*	*Bladder (cc)*	*Overlap PTV & Bladder*
Pt1	1PRI	113.8	69.3	11.0%	284.3	2.7%
	2BST	65.0	69.3	3.4%	284.3	0.8%
Pt2	1PRI	196.7	79.7	7.5%	273.4	6.6%
	2BST	129.5	79.7	4.1%	273.4	3.9%
Pt3	1PRI	155.8	48.8	2.5%	462.5	5.1%
Pt4	1PRI	83.8	92.5	3.0%	443.5	1.0%
Pt5	1PRI	118.9	63.0	8.6%	267.8	6.8%
	2BST	67.9	63.0	3.8%	267.8	2.4%
Pt6	1PRI	171.0	65.7	10.2%	345.2	5.1%
	2BST	90.4	65.7	6.6%	345.2	1.6%
Pt7	1PRI	183.0	47.8	5.4%	295.7	5.0%
	2BST	83.7	47.8	5.2%	295.7	1.6%
Pt8	1PRI	90.9	69.0	4.7%	145.3	2.9%
Pt9	1PRI	104.2	69.3	9.7%	123.8	7.2%
	2BST	54.3	69.3	4.6%	123.8	4.2%
Pt10	1PRI	94.6	85.1	1.7%	102.8	6.3%

### A. Expert Plan and Clinical Plan

Expert Plan started with the conventional trial‐and‐error approach with an institutional OAR objective template modified from Radiation Therapy Oncology Group (RTOG) guidelines.^(13)^ The institutional dose‐volume constraints used in this study are: $D_{25\%}<65\;\text{Gy}$, $D_{40\%}<40\;\text{Gy}$ for bladder, and $D_{17\%}<65\;\text{Gy}$, $D_{40\%}<40\;\text{Gy}$ Gy for rectum, and $D_{\text{max}}<50\;\text{Gy}$ for femoral heads. The maximum PTV dose must not exceed 110% of prescription dose, with <105% preferred. To promote PTV dose homogeneity, maximum dose of 102% and minimum dose of 98% of the prescription were used as starting PTV objectives during optimization.

The institutional constraints were used as a starting point for the Expert Plan. The planner made on‐the‐fly adjustments to the starting objectives. This trial‐and‐error process continued until 150 iterations were reached. This plan with 150 interactions is referred to as Expert Plan. After 150 iterations, the planner made a decision whether to continue adjusting OAR objectives based on personal knowledge and experience. This process would continue until the planner is satisfied of the dosimetry. This final plan is referred as the Clinical Plan.

The Expert Plan and Clinical plans are referred as the two types of manual plans. While there is a 150 iteration limitation for the Expert Plan, there is no time limitation for the Clinical plan, which better represents the clinical IMRT planning practice. The entire planning process was recorded using BB FlashBack software to analyze optimization time and planning efficiency.

### B. Automatic Plan (PAF Plan)

The automatic plan, subsequently referred to as the PAF Plan, uses fixed patient‐specific objective settings, as opposed to adjustable objectives used in Expert/Clinical Plans. This set of fixed patient‐specific objectives was predetermined from the PAF model, which was derived from more than 100 previous clinically approved prostate IMRT plans.^(12)^ The PAF‐predicted DVH objectives were based on past clinically approved plans and reflected the best available clinical planning experience. To obtain an objective set for a particular patient, the patient's anatomical information was firstly exported into an in‐house MATLAB (MathWorks, Natick, MA) program. The dose volume objectives were generated using the PAF model for PTV, bladder, rectum, and femoral heads. These PAF objectives were then imported to the treatment planning system, replacing the institutional objective template at the beginning of the optimization. Since there is no additional adjustment to these objectives throughout the optimization, the process is considered fully automatic. To be comparable with the expert planning, the maximum iteration was also set to 150 for the PAF plan. The entire planning process was also recorded using BB FlashBack software.

### C. Plan evaluation

The quality of Expert, Clinical, and PAF Plans was evaluated by comparing DVH parameters. The $D_{\text{MAX}}$ and $D_{98\%}$ for PTV, $D_{\text{MAX}}$, D_10cc_, $D_{25\%}$, and $D_{40\%}$ for bladder, $D_{\text{MAX}}$, D_10cc_, $D_{17\%}$, and $D_{40\%}$ for rectum, and $D_{\text{MAX}}$ for femoral heads were selected as key parameters for detailed analysis. All dosimetric parameters were compared in percentage of prescription dose.

In addition to dosimetric quality, the number of objective adjustments made by the planner during optimization was compared to evaluate planning effort and efficiency. For PAF Plans, the optimization finished at 150 iterations with no user adjustment. For Expert and Clinical Plans, the amount of adjustments reflected the efforts and efficiencies from the planner. The more efforts required, the less efficient is the optimization process.

## III. RESULTS

### A. Planning quality comparison

All the Expert, Clinical, and PAF Plans satisfied the basic clinical standard as they all met the institutional objectives for bladder, rectum, and femoral heads. With the same normalization that ensures prescription dose covers at least 95% of the PTV, the PTV $D_{\text{MAX}}$ was <110% for all plans. The $D_{98\%}$ of PTV for PAF Plans was 0.82% higher than Expert and Clinical Plans, on average, with a maximum of 2.31% and 1.96% higher, respectively.

**Figure 2:**
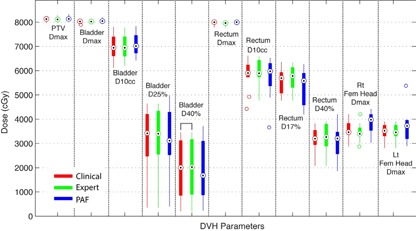
Box plot for dosimetric comparisons between Expert, Clinical, and PAF Plans. The key parameters include $D_{\text{MAX}}$ for PTV, $D_{\text{MAX}}$, D_10cc_, $D_{25\%}$, and $D_{40\%}$ for bladder, $D_{\text{MAX}}$, D_10cc_, $D_{17\%}$, and $D_{40\%}$ for rectum, and $D_{\text{MAX}}$ for femoral heads. Wilcoxon signed‐rank test indicates that, for all DVH parameters, there is no significant difference between three groups of plans.

A box plot of the dosimetric parameters for all three groups of plans is shown in Fig. 2. Even though the planner decided further optimization and extra adjustments of objectives would improve the dosimetric quality, the Clinical Plans only offered very limited benefits in low/mid‐dose regions of OARs and were in general slightly worse in high‐dose regions, when compared to Expert Plans. The differences for $D_{\text{MAX}}$ of PTV, bladder, and rectum between Expert and Clinical plans were <0.51%, on average, with 2.07% maximum higher for Clinical Plans. Bladder D_10cc_, $D_{25\%}$, and $D_{40\%}$ were in general lower for Clinical Plans, but <0.49% on average. Rectum D_10cc_, $D_{17\%}$, and $D_{40\%}$ for Clinical Plans were also 1.43%, 1.35%, and 1.02% lower, on average, than Expert Plans, with maximum differences of 11.65%, 10.18%, and 11.21%, respectively. $D_{\text{MAX}}$ for femoral heads were comparable (<35 Gy on average) for both Expert and Clinical Plans.

The dosimetric parameters of PAF Plans were also compared to those of Clinical Plans. As shown in Fig. 2, in general, the differences between PAF Plans and Clinical Plans were small across all dosimetric parameters. Relative to prescription doses, the $D_{\text{MAX}}$ of PTV and rectum were 0.06% (−1.74% to 2.65%) and 0.14% (−1.56% to 2.20%) lower, while $D_{\text{MAX}}$ of bladder was 0.46% (−1.63% to 2.46%) higher, on average, for PAF plans. Bladder D_10cc_ for PAF Plans was in general higher than Clinical Plans, but <1.54%. Bladder $D_{25\%}$ and $D_{40\%}$ for PAF Plans were lower than Clinical Plans, by on average 0.69% (−7.71% to 4.57%) and 0.81% (−6.81% to 4.04%), respectively. Rectum D_10cc_, $D_{17\%}$, and $D_{40\%}$ for PAF Plans were also 2.11%, 2.72%, and 0.27% lower, on average, than Clinical Plans, and up to 10.10%, 10.07%, and 8.69%, respectively. $D_{\text{MAX}}$ for femoral heads were comparable for both PAF Plans and Clinical Plans (<35 Gy on average).

Wilcoxon signed‐rank test^(14)^ on Expert–Clinical and PAF–Clinical Plan pairs showed no statistical difference for all dosimetric parameters, except bladder $D_{40\%}$ for Expert–Clinical Plan pair, but the absolute dose difference was only −0.4%±0.9% across all patients. Examples of dose distributions and DVHs for Expert, Clinical, and PAF Plans are shown in 3, 4.

**Figure 3:**
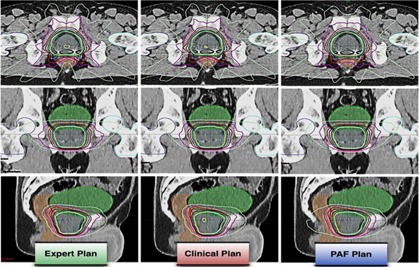
Example dose distributions for Expert, Clinical, and PAF Plans. The three groups of plans are comparable, while the Clinical Plans can provide slightly better rectum sparing in the lower/medium dose region.

**Figure 4:**
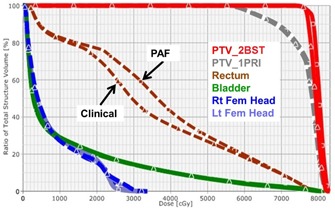
Example DVH comparisons for Clinical and PAF Plans showing that the Clinical and PAF Plans were comparable. They are almost the same for PTV, bladder, and femoral heads. High‐dose region is also similar for rectum, while Clinical Plans may provide slightly better sparing in the low/medium‐dose region.

### B. Effort and efficiency comparison

The optimization for PAF, Expert, and Clinical Plans was finished within 3.2 (2.1 to 4.5), 4.0 (3.3 to 4.9), and 5.9 (3.3 to 9.1) min, on average, for primary plans and 2.0 (1.4 to 2.3), 3.6 (2.9 to 4.4), and 4.9 (3.1 to 7.2) min for boost plans, respectively, on an workstation with a 6‐core Xeon processor and 24 GB memory. Compared to Clinical Plan (Primary+Boost), the average optimization time for PAF Plan was reduced by 5.2 min on average, with a maximum time reduction of 7.1 min. In terms of delivery efficiency, the PAF Plans required 130 and 107 fewer monitor units (MU), on average, compared to the Clinical Plans for the primary (794 to 1154 MUs) and boost (574 to 738 MUs) plans, respectively. The likely reason for this efficiency gain is that the PAF‐predicted DVH represented a good balancing between PTV coverage and OARs sparing, and thus were able to produce smoother fluence than the manual optimization. While during the manual optimization process, the multiple back‐and‐forth adjustments of objectives could cause fluence over‐modulation, and therefore required more MUs to deliver the same dose.

In addition to total optimization time comparison, the amounts of user adjustments were also compared among different planning methods. To achieve the Clinical Plans, the human planner had to adjust dose volume objectives on average by 4 to 14, 11 to 33, and 2 to 6 times for bladder, rectum, and femoral heads, respectively. Among all the OARs, the objectives for bladder and rectum were mostly adjusted by the planner. During the adjustments, dose and volume objectives were lowered by up to 75% and 37% from initial values set at the beginning of the optimization, based on institutional template. In addition to DVH objectives, the objective priorities were adjusted multiple times for bladder and rectum by up to 20%. The PTV objective priority was also adjusted for every case and by ∼10%.

## IV. DISCUSSION

In this study, a PAF model‐driven automatic IMRT plan optimization process was compared to two manual optimizing scenarios: under time constraint and with unlimited optimization time. The results showed highly comparable dosimetry quality with substantial benefits in planning efficiency. To achieve similar levels of target coverage and OAR sparing, the PAF Plan did not involve any user adjustment, compared to the Expert and Clinical Plans. The PAF plan also provided better delivery efficiency; average total MU in PAF Plans were ∼100 fewer than those in Expert and Clinical Plans.

Although the saving of planning time by 5 min may not be clinically significant for a single prostate IMRT plan in a light workload environment, it is unsupervised and can be executed without human intervention. For batch plan generation, trade‐off evaluation, or heavy load clinics, such saving of planning resources could be valuable. We are in the process of evaluation the efficiency gain on head and neck planning tasks, which is much more complicated and is expected to benefit more from this PAF‐based automated planning workflow.

It is important to note that the purpose of this study is to evaluate the performance of the PAF‐based automatic planning system developed at our institution by comparing the plan quality to Expert and Clinical Plans. Evaluation studies of the similar nature have been conducted by other groups,^10^, ^15^ but for their particular knowledge‐based planning implementation only, which is different from what was used in this study.

Currently, the Expert and Clinical Plans were performed by one single planner. The clinical planning efforts could vary substantially among planners, as a result of planners' experiences with IMRT planning, and also due to different optimization packages.^(16)^ Another factor to note is that both the expert and clinical planning were video‐recorded (see Appendix A: IMRT Planning Demo Video, uploaded separately to the website) and the planner tended to adjust as quickly as possible, even though that was not the intention. So in reality, the Clinical Plans may need more adjustments than shown in this study. It should also be noted that the efforts and efficiencies compared in this study are for optimization only and should not be confused with the total planning reported in other studies.^(16)^ In clinical treatment planning, the optimization is only one of the many steps needed to produce a clinically deliverable plan, besides contouring, chart preparation, and QA.

From our observation, only three out of ten primary and two out of six boost Expert Plans were considered “optimal” by the planner at 150 iterations. All the other Expert Plans were further adjusted based on the judgment of the planner, under the expectation that a better OAR sparing and/or target coverage could be achieved. However, quantitative comparison between Expert Plans (without additional adjustment) and Clinical Plans (with additional adjustments) shows that the actual benefits of further adjustments after 150 iterations were very limited — <1% difference on average. This indicates that, due to lack of knowledge of achievable DVH for a particular anatomy, the planner does not exactly know when to adjust, where to adjust, and even when to stop optimization. In other words, even an experienced planner does not know whether an optimal plan has been achieved without clear knowledge on the correlation between anatomy features and achievable DVH. A planner has to rely on the additional adjustments till no further improvement can be achieved, which is considered inefficient. On the other hand, the PAF‐based automatic planning has learned this anatomy‐DVH correlation during model training process based on a large number of high‐quality clinical plan samples, and can therefore more accurately predict the achievable DVH for a particular anatomy under institutional plan quality consensus with less variation than individual planner's judgment. Therefore the objectives acquired through PAF modeling can offer more accurate and efficient guidance for the optimizer to achieve a clinical‐quality plan.

**Figure 5:**
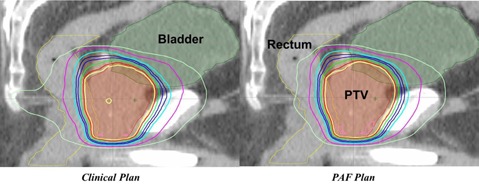
An example when PAF‐based objectives helped to achieve better OAR sparing. In this case, the PAF Plan further reduced rectum dose by 7.7 Gy for both $D_{10\text{cc}}$ and $D_{17\%}$ compared to the Clinical Plan.

In addition to improvement in optimization efficiency and quality consistency, PAF‐based DVH objectives helped achieve better OAR dose reduction for some cases. For one case among the ten randomly selected patients as shown in Fig. 5, the PAF plan achieves reduction in rectum D_10cc_, $D_{17\%}$, and $D_{40\%}$ by 7.7 Gy, 7.7 Gy, and 4.6 Gy, when compared to the Clinical Plan. Detailed evaluation shows that the Clinical Plan was already far below the institutional objectives constraints, and was considered clinically acceptable. In cases like this, even for an experienced planner, it was still difficult to precisely estimate whether the optimal sparing has been achieved. Since the planner did not know the achievable DVH for this specific patient at the time of planning, further reduction in OAR dose was not pursued. PAF‐based objectives, on the other hand, were able to observe such favorable anatomical feature, and predict a lower DVH based on patient anatomy before the optimization, guiding the optimizer towards more — but still reasonable — OAR sparing improvement.

During the planning process, all PAF‐modeled DVH objectives were met within the optimizer. However, the final DVHs after volume dose calculation show some deviations from the corresponding PAF objectives for some cases. This is due to the difference between dose calculation algorithms used by the optimizer and the volume dose calculation. The multiresolution dose calculation (MRDC)^(17)^ algorithm used in the optimizer sacrifices the calculation accuracy for speed with less accurate source modeling and no heterogeneity correction for secondary radiations. On the other hand, the volume dose calculation performed after optimization uses analytic anisotropic algorithm (AAA),^(17)^ which has more accurate primary photon, secondary photon, and electron contamination modeling, as well as heterogeneity correction. Therefore the DVH shown in the optimizer could be different from final plan DVH calculated by the more accurate algorithm, even though they are calculated based on the same set of fluence maps. This difference is more pronounced in cases with more heterogeneity (such as large volume of rectal gas) and interferes with the PAF's guidance to the optimizer. Even if all PAF objectives are met during the optimization, the final DVH might still not fully match the predicted DVH. This issue is not a limitation of the PAF modeling, but of the current treatment planning system that we are using, and could be potentially alleviated or resolved by the intermediate dose calculation feature introduced in the newer version of treatment planning system.

In addition to dose calculation, large heterogeneity could also result in reduced prediction accuracy. The PAF models were built based on modeling the anatomy and dose relationship of clinical cases that mostly had empty rectum,^(12)^ which is in accordance with our clinical practice guidelines. The PAF model does not differentiate rectal gas from tissue. For a case with large rectal gas, the PAF model predicted the objectives based on the outer contour of an empty rectum without considering the air cavity inside, whereas in reality the presence of large air cavity would change the dose distribution in the volume dose calculation. Therefore in cases like this, the objectives generated from PAF model were somewhat inaccurate, and could lead to suboptimal plans. We are currently investigating solutions to overcome this limitation.

The PAF model does not predict the optimal number of beams or optimal beam angle. In this study, a universal beam arrangement of 25°, 70°, 130°, 180°, 230°, 290°, and 335° and 40°, 110°, 180°, 250°, and 320° was employed for the primary and boost treatments, respectively. Since our institution uses a beam set of template for prostate IMRT plans, the fixed beams angle selection is consistent with our clinical practice.

## V. CONCLUSIONS

In this study, the knowledge‐guided automatic IMRT optimization was compared to manual optimization based on both dosimetry and efficiency for prostate cancer. The results show that the plans generated automatically based on the PAF knowledge model easily met the institutional or RTOG objectives for OARs, and are comparable to the manual plans in key DVH parameters of PTV and OARs. In addition, the PAF process substantially reduces optimization efforts, and is independent of planner's knowledge and experience. Therefore, the PAF‐based automatic IMRT treatment planning may bring positive clinical impact if implemented in the clinical treatment planning system, and might enable automatic IMRT planning without or with minimum human intervention.

## ACKNOWLEDGMENTS

This research was partially supported by a research grant from NIH/NCI 1R21CA161389 and a master research grant from Varian Medical Systems.

## APPENDIX A: IMRT Planning Demo Video

(see file uploaded separately to the website)

## Supporting information

Supplementary Material

## References

[1] Berger J. ROENTGEN: case‐based reasoning and radiation therapy planning. Proceedings of the Annual Symposium on Computer Application in Medical Care. 1992:210–14. PMC2248035 1482869

[2] Chen S , Zhou S , Yin FF , Marks LB , Das SK . Investigation of the support vector machine algorithm to predict lung radiation‐induced pneumonitis. Med Phys. 2007;34(10):3808–14. 17985626 10.1118/1.2776669 PMC2920285

[3] Chen S , Zhou S , Zhang J , Yin FF , Marks LB , Das SK . A neural network model to predict lung radiation‐induced pneumonitis. Med Phys. 2007;34(9):3420–27. 17926943 10.1118/1.2759601 PMC2721274

[4] El Naqa I , Suneja G , Lindsay PE , et al. Dose response explorer: an integrated open‐source tool for exploring and modelling radiotherapy dose‐volume outcome relationships. Phys Med Biol. 2006;51(22):5719–35. 17068361 10.1088/0031-9155/51/22/001

[5] Gulliford SL , Webb S , Rowbottom CG , Corne DW , Dearnaley DP . Use of artificial neural networks to predict biological outcomes for patients receiving radical radiotherapy of the prostate. Radioth Oncol. 2004;71(1):3–12. 10.1016/j.radonc.2003.03.001 15066290

[6] Moore KL , Brame RS , Low DA , Mutic S . Experience‐based quality control of clinical intensity‐modulated radiotherapy planning,” Int J Radiat Oncol Biol Phys. 2011;81(2):545–51. 21277097 10.1016/j.ijrobp.2010.11.030

[7] Moore KL , Brame RS , Low DA , Mutic S . Quantitative metrics for assessing plan quality. Sem Radiat Oncol. 2012;22(1):62–69. 10.1016/j.semradonc.2011.09.005 22177879

[8] Wu B , Ricchetti F , Sanguineti G , et al. Patient geometry‐driven information retrieval for IMRT treatment plan quality control. Med Phys. 2009;36(12):5497–505. 20095262 10.1118/1.3253464

[9] Zhu X , Ge Y , Li T , Thongphiew D , Yin FF , Wu QJ . A planning quality evaluation tool for prostate adaptive IMRT based on machine learning. Med Phys. 2011;38(2):719–26. 21452709 10.1118/1.3539749

[10] Good D , Lo J , Lee WR , Wu QJ , Yin FF , Das SK . A knowledge‐based approach to improving and homogenizing intensity modulated radiation therapy planning quality among treatment centers: an example application to prostate cancer planning. Int J Radiat Oncol Biol Phys. 2013;87(1):176–81. 23623460 10.1016/j.ijrobp.2013.03.015

[11] Appenzoller LM , Michalski JM , Thorstad WL , Mutic WL , Moore KL . Predicting dose‐volume histograms for organs‐at‐risk in IMRT planning. Med Phys. 2012;39(12):7446–61. 23231294 10.1118/1.4761864

[12] Yuan L , Ge Y , Lee WR , Yin FF , Kirkpatrick JP , Wu QJ . Quantitative analysis of the factors which affect the interpatient organ‐at‐risk dose sparing variation in IMRT plans. Med Phys. 2012;39(11):6868–78. 23127079 10.1118/1.4757927

[13] RTOG 0815 – a Phase III prospective randomized trial of dose‐escalated radiotherapy with or without short‐term androgen deprivation therapy for patients with intermediate‐risk prostate cancer, Pittsfiled, MA: Berkshire Health Systems; 2012.

[14] Wilcoxon F . Individual comparisons by ranking methods. Biometr Bull. 1945;1(6):80–83.

[15] Zarepisheh M , Long T , Li N , et al. A DVH‐guided IMRT optimization algorithm for automatic treatment planning and adaptive radiotherapy replanning. Med Phys. 2014;41(6):061711. 24877806 10.1118/1.4875700

[16] Das IJ , Moskvin V , Johnstone PA . Analysis of treatment planning time among systems and planners for intensity‐modulated radiation therapy. J Am Coll Radiol. 2009;6(7):514–17. 19560069 10.1016/j.jacr.2008.12.013

[17] Varian Medical Systems . Eclispe algorithm reference guide. Palo Alto, CA: Varian Medical Systems Inc.; 2012.

